# Intestinal Spirochetosis: Do We Always Treat It or Is It a Self-Limiting Disease?

**DOI:** 10.4103/1319-3767.49010

**Published:** 2009-04

**Authors:** Apurva Sinha, Tony Mak, Shabina Petkar, Arthur Allan

**Affiliations:** Department of Colorectal Surgery, Good Hope Hospital, Sutton Coldfield, Birmingham B75 7RR, UK. Email: apurv2000@yahoo.com

Sir,

A 54-year-old man was referred by his GP for intermittent, painless self-limiting episode of rectal bleeding of 6-months duration. Patient denied drug abuse and there was no history of homosexuality.

During his first clinic presentation, the physical examination was unremarkable except on his rigid sigmoidoscopy, we could find a small suspicious neoplastic looking like lesion 8 cm from anal margin, and thus biopsy was taken and sent for histopathology. He also had large hemorrhoids, which were banded during the same appointment. Stool examinations revealed no conventional pathogens. A full blood count and biochemistry determinations were normal.

His initial biopsy report showed active chronic inflammation but indeterminate in type. It was, therefore, concluded that this was likely to be dysplasia-associated lesion or mass associated with inflammatory bowl disease.

He underwent follow-up flexi-sigmoidoscopic biopsy, which was reported as being of inflammatory bowel disease, more likely to be of Crohn's disease. The rest of the colon was reported as normal in a follow up barium enema.

**Figure 1:**
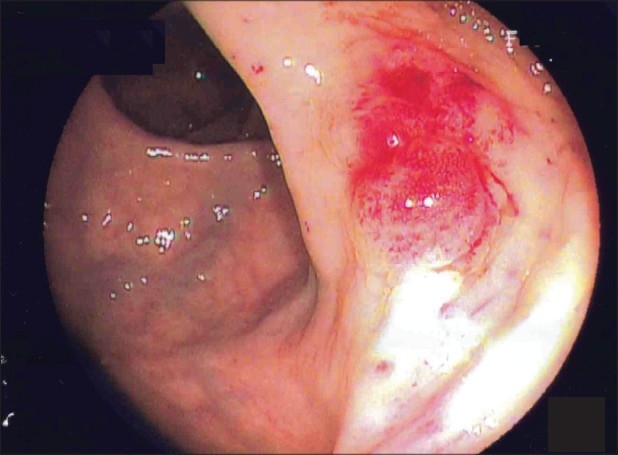
Endoscopic image showing lesion found in the rectum at 10 o'clock position

**Figure 2:**
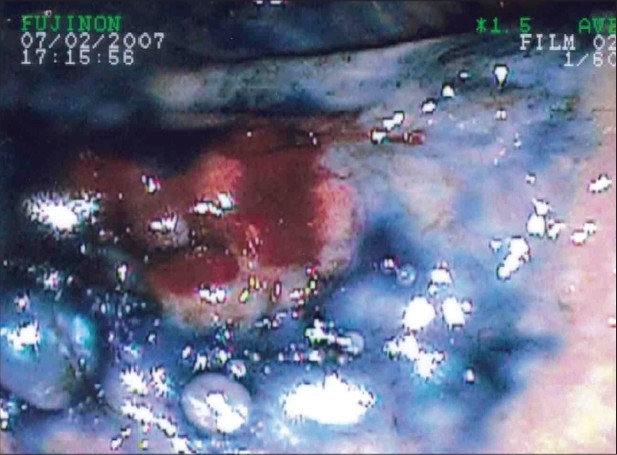
Endoscopic image after dye spray of the area which didnot take up dye

He remained asymptomatic after his initial banding. He was again followed up for routine check flexi-sigmiodoscopy [Figure 1, Figure 2], and histology from this biopsy was suggestive of intestinal spirochetosis, but with no evidence of any acute or chronic inflammation.

Clinically, patient remained asymptomatic with no symptoms suggestive of any fresh episode of rectal bleeding or proctitis.

Intestinal spirochete infection is common in commercial poultry flocks as shown by studies in Australia.[1]

The prevalence of spirochetosis in human varies from 2.5 to 16% in western countries.[2] The prevalence in homosexual and immune-compromised patients, based on stool culture and biopsy[3] finding was as high as 50%.

In human, the pathological and clinical significance of these organisms is far less clear and controversial although there have been reported cases associated with rectal bleeding and diarrhea.[4]

The histological appearance of this organism is that of a ***false brush border*** on the colonic mucosa, which represents a layer of spirochetes, especially ***Brachyspira aalborgi*** and ***Brachyspira pilosicoli*** (***Serpulina pilosicoli***).

It can be identified readily on H and E-stained sections although silver stains also can be used to highlight the organisms and can detect cases not evident on routine H and E stains.[3] It is also important to note that these organisms are difficult to grow on ordinary culture medium, thus making characterization difficult in clinical practice.

The most appropriate means of identification is by using polymerse chain reaction-based technique, is the best way of diagnosing the disease,[5] which is sophisticated, not easily available and costly, especially in developing countries. This may be the cause of it being under-reported and overlooked by less experienced surgical pathologists.[5]

In this case, it might be argued that it is one of the incidental findings in an asymptomatic patient and thus required no active treatment. But in symptomatic and high-risk group an active treatment with metronidazole is needed. It is also important to consider patient symptoms and risk category before starting the treatment.

Patient symptoms and risk category are paramount in identifying cases. Spirochetosis, therefore, should be looked into all rectal biopsy specimens, especially in high-risk and symptomatic patients. In an asymptomatic patient, it should be treated as a self-limiting disease.
